# Cervical cancer mortality in Peru: regional trend analysis from 2008–2017

**DOI:** 10.1186/s12889-021-10274-1

**Published:** 2021-01-26

**Authors:** J. Smith Torres-Roman, Luz Ronceros-Cardenas, Bryan Valcarcel, Miguel A. Arce-Huamani, Janina Bazalar-Palacios, Jorge Ybaseta-Medina, Carlo La Vecchia, Christian S. Alvarez

**Affiliations:** 1 Universidad Científica del Sur, Lima, Peru; 2 Latin American Network for Cancer Research (LAN–CANCER), Lima, Peru; 3 Universidad Católica Los Ángeles de Chimbote, Instituto de Investigación, Chimbote, Peru; 4 Facultad de Medicina Humana, Universidad Nacional San Luis Gonzaga, Ica, Peru; 5 Department of Clinical Sciences and Community Health, Università degli Studi di Milano, 20133 Milan, Italy; 6 Division of Cancer Epidemiology and Genetics, National Cancer Institute, Rockville, MD USA

**Keywords:** Cervical cancer, Mortality, Trends, Epidemiology, Indigenous population, Peru

## Abstract

**Background:**

Cervical cancer is the third leading cause of cancer-related death among Latin American women. Peru has the sixth highest mortality rate for cervical cancer in the region with regional variations. We aimed to determine overall and regional cervical cancer mortality rates and trends in Peru between 2008 and 2017.

**Methods:**

We performed an ecological study on the number of deaths by cervical cancer in Peru. Deaths were extracted from the Peruvian Ministry of Health mortality database. Age-standardized mortality rates (ASMR) were estimated per 100,000 women-years using the world standard Segi population. We computed mortality trends using the Joinpoint regression program, estimating the annual percent change (APC). For spatial analysis, GeoDA software was used.

**Results:**

Peru showed downward trends in the last decade (from 11.62 in 2008 to 9.69 in 2017 (APC = − 2.2, 95% CI: − 4.3, − 0.1, *p* < 0.05). According to regional-specific analysis, the highest ASMR was in the rainforest region, although this declined from 34.16 in 2008 to 17.98 in 2017 (APC = − 4.3, 95% CI: − 7.2, − 1.3, *p* < 0.01). Concerning spatial analysis and clustering, the mortality rates from 2008 to 2017 showed a positive spatial autocorrelation and significant clustering (Moran’s I: 0.35, *p* < 0.001) predominantly in the neighboring North-East departments (Loreto, Ucayali, and San Martin).

**Conclusions:**

Although mortality trends in the entire population are decreasing, mortality rates remain very high, mainly in the rainforest region. Our results encourage a need for further development and improvement of the current health care delivery system in Peru.

## Background

Cervical cancer is the fourth leading cause of cancer-related mortality among women worldwide [1] and the third in Latin America [2]. In 2018, GLOBOCAN reported an age-standardized mortality rate (ASMR) of 7.1/100,000 women-years in South America, which is significantly higher compared to other regions such as Western Europe (2.1), North America (1.9), or Australia/New Zealand (1.7) [1, 3]. The highest mortality rates in Latin America were reported in Bolivia (21.0), Nicaragua (18.3), and Paraguay (15.7) [2].

The Pan American Health Organization (PAHO) has described that the variations of cervical cancer mortality rates among Latin American countries that correlate with the inequalities in each country [4]. Geographic location, access to health care, knowledge, education, cultural behaviors, and socioeconomic status are among the most prominent barriers for reducing mortality by cervical cancer [4]. In Peru, cervical cancer is the leading cause of death by cancer among women of reproductive age [5]. The cervical cancer rates in the coastal region are substantially lower than other parts of the country due to higher socioeconomic status, and increased access to screening and specialized care [6]. Moreover, these rate disparities have important implications in Peru, where the uptake of early screening via the Pap smear (Pap) test is low [7], particularly in women living in rural areas [8].

The most recent governmental strategy called the “Plan Esperanza” [9], aims to decentralize health care delivery and reduce out-of-pocket expenditure for Peruvian citizens with low economic income and those living in vulnerable settings [9]. On the other hand, private health insurance provides cancer appointments and free screening to insurers [6].

Despite the efforts of Plan Esperanza to provide comprehensive coverage of cervical cancer screening, treatment, and medical care, this strategy is still restricted to urban areas [10]. Given the high and variability of the mortality rates among the Peruvian departments, we aimed to examine the overall and regional trends of cervical cancer mortality in Peru between 2008 and 2017. We further analyzed regional mortality trends in Peru.

## Methods

### Design and study setting

We performed an ecological study based on secondary data analysis using the Peruvian Ministry of Health (Known by its Spanish acronym, MINSA) mortality database. The data for mortality by cervical cancer was obtained from 2008 to 2017. Cervical cancer was identified by the code C53 according to the International Classification of Disease – 10th edition (ICD-10) [11]. MINSA is responsible for collecting mortality data from administrative records of private and public health facilities across the 25 departments of Peru. The information is available upon request on the MINSA online platform: http://www.minsa.gob.pe/portada/transparencia/solicitud/.

MINSA collects mortality data at the national level from different sources, including all health establishment records, the National Registry of Identification and Civil Status*,* and the Public Ministry of Health [12]. The data collected include the number of deaths for each disease aggregated by 5-year sex-specific age groups [12–14].

Peru is in the Andean region of South America and has a population of 31 million inhabitants distributed among three regions: coastal, highlands, and rainforest [15]. The coastal region spans 11.7% of the national territory, but it is the most populated region with approximately 56.3% of the total population of Peru. The highlands cover approximately 27.9% of the national territory and includes 29.7% of the population. Lastly, the rainforest is the largest region of the country, which accounts for 60.3% of the national territory but only contains 14% of the total population [15].

### Ethical considerations

The current study utilized de-identified, publicly available data; therefore, ethical approval is not required. In order to obtain the raw data, an online form was completed through: https://www.minsa.gob.pe/portada/transparencia/solicitud/.

### Correction of under-reporting

The cause of death notification system throughout Peru is omissive [16]. The underreporting of deaths was corrected by year and per department of those registered by the Ministry of Health and death estimations by the National Statistics Institute (NSI) [16]. The variability coverage of the 2008 to 2017 registry of deaths was correct to determine the underreporting rate as reported in previous studies [14, 17] using the following formula:

$$ \mathrm{R}=100-\left(\mathrm{OD}/\mathrm{ED}\right)\times 100 $$

R = Underreporting rate

OD = number of deaths observed in each department.

ED = number of deaths estimated in each department.

### Statistical analysis

After correcting the number of deaths, the age-standardized mortality rates (ASMR) were estimated per 100,000 women-years and adjusted to the world standard SEGI population [18] . The population denominators were obtained from census data in 2010, and 2015 conducted by NSI, which is responsible for regulating, planning, directing, coordinating, and supervising the official statistics of the country [19]. We examined the overall and regional mortality trends using the Joinpoint regression program version 4.7.0 [20], which identified significant trend change points and the rate of change (annual percent change [APC]) [21]. The significance levels utilized were based on the Monte Carlo permutation model and the calculation of the APC ratio utilizing the logarithm of the ratio [22, 23]. Additional analyses were performed to evaluate the overall, regional cervical cancer mortality trends pre-(2008–2011), and post-implementation (2012–2017) of Plan Esperanza.

GeoDA software was employed for the spatial analysis. We computed the highest and lowest ASMR of cervical cancer in the neighboring departments [24]. The map showed a spatial typology consisting of five categories as in previous studies in Peru [13, 15]: (i) (‘high–high’), (ii) (‘low–high’), (iii) (‘low–low’), (iv) (‘high–low’), and (v) ‘not significant’. The Moran’s I values ranged from − 1 to + 1. We used a reference distribution using 999 random permutations to indicate statistical significance (*p* < 0.05) [24, 25].

## Results

Over 10 years, we identified an annual average of 990 deaths from cervical cancer, of which increased to 1729 deaths after correcting for underreporting of deaths. Similarly, the uncorrected mortality rates per 100,000 women-years increased from 6.8 to 11.8 in the entire population. When analyzed by region, the rainforest area showed the highest corrected average mortality rate (26.3 per 100,000 women-years), followed by the highlands (14.2 per 100,000 women-years), and the coastal region (9.5 per 100,000 women-years) (Table 1).

**Table 1:** Cervical cancer deaths and mortality rates in Peru according to geographical area from 2008 to 2017

Geographical areas	Annual deaths^**a**^		Mortality rates^**b**^	
	Uncorrected	Corrected	Uncorrected	Corrected
**Coast region**	654	942	6.6	9.5
Ancash	25	54	4.6	9.8
Arequipa	41	60	6.2	8.9
Callao	36	43	6.7	8.2
Ica	24	27	6.2	7.1
La Libertad	73	100	8.4	11.6
Lambayeque	77	95	12.1	15.0
Lima	304	451	5.9	8.7
Moquegua	9	13	10.3	16.0
Piura	50	75	6.1	9.3
Tacna	14	20	9.2	13.7
Tumbes	2	4	2.5	4.8
**Highlands region**	248	511	6.9	14.2
Apurimac	8	20	4.1	10.2
Ayacucho	14	34	5.0	12.4
Cajamarca	32	81	5.0	12.6
Cusco	29	68	4.8	11.2
Huancavelica	12	25	6.8	14.0
Huanuco	49	100	13.7	27.8
Junin	63	108	11.0	18.6
Pasco	10	21	9.0	19.5
Puno	31	55	4.9	8.6
**Rainforest region**	87	275	8.4	26.3
Amazonas	8	29	4.8	18.5
Loreto	28	136	7.8	37.4
Madre de Dios	8	10	24.0	30.2
San Martin	21	58	7.5	20.2
Ucayali	23	43	11.4	21.4
**Peru**	990	1729	6.8	11.8

^a^ Annual average of deaths for a 10-year period (2008–2017)

^b^ Age-standardized rates per 100,000 women-years

**Fig. 1:**
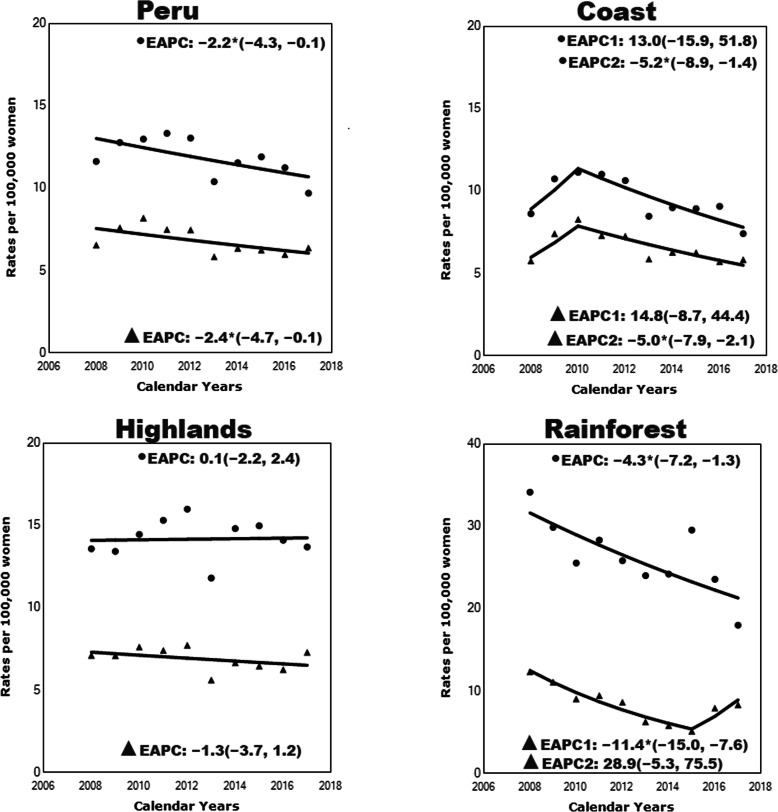
Joinpoint regression analysis of uncorrected (▲) and corrected (●)mortality rates in Peru and its regions from 2008 to 2017

Figure 1 displays the results of the joinpoint regression analysis for the uncorrected and corrected mortality rates in Peru and its’ regions between 2008 and 2017. For the corrected rates, Peru experienced a significant downward trend from 11.62 in 2008 to 9.69 per 100,000 women-years in 2017 (APC = − 2.2, 95% confidence interval [CI]: − 4.3, − 0.1, *p* < 0.05). Similarly, the coast and the rainforest region showed downward trends in cervical cancer mortality. The coast started a decreasing trend in 2010 (APC = − 5.2, 95% CI: − 8.9, − 1.4, *p* < 0.05), while the rainforest showed a reduction amongst the entire study period (APC = − 4.3, 95% CI: − 7.2, − 1.3, *p* < 0.05). The highlands were the only region with non-significant trends (APC = 0.1, 95% CI: − 2.2, 2.4, *p* = 0.07) (Fig. 1).

**Fig. 2:**
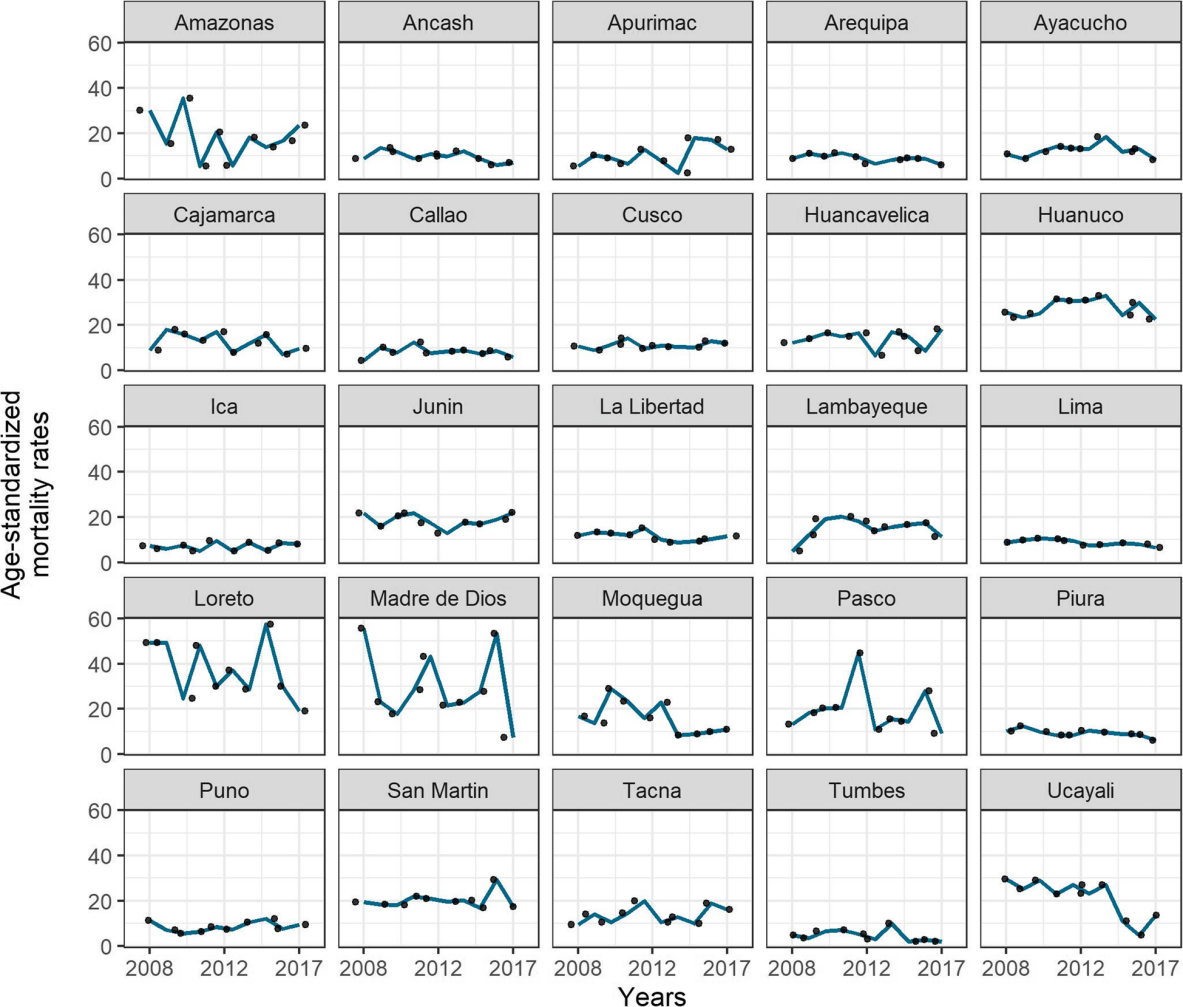
Mortality trends in Peruvian departments between 2008 and 2017

Table 2 shows the cervical cancer mortality rates (per 100,000 women-years), the percent change, and the estimated APC among Peruvian women between 2008 and 2017 in each geographical area. Three coastal departments showed significant downward trends: Arequipa, from 8.82 to 6.10 (31% reduction; APC = − 3.9; 95% CI: − 8.1, 0.4; *p* < 0.05); Lima, from 8.66 to 6.58 (24% reduction; APC = − 3.6, 95% CI: − 6.3, − 0.8; *p* < 0.05); and Piura, from 10.26 to 5.97 (42% reduction; APC = − 4.3, 95% CI: − 7.7, − 0.7; *p* < 0.05). In addition, Ucayali was the only department of the rainforest region that showed a significant reduction in the mortality rate from 29.53 in 2008 to 13.60 in 2017 (54% reduction; APC = − 12.9, 95% CI: − 21.8, − 3.2; *p* < 0.05). No significant downward trends were observed in the highland departments (Table 2 and Fig. 2).

**Table 2:** Cervical cancer mortality rates (per 100,000 women-years), data availability, percent change, and estimated annual percent change and the corresponding 95% confidence intervals in Peruvian women between 2008 and 2017

Geographical areas	Cervical cancer mortality rates (per 100,000 women-years)				
	2008	2017	% change (2017/2008)	EAPC	95%CI
**Coast region**					
Ancash	8.85	7.05	−20	− 4.9	−9.8, 0.2
Arequipa	8.82	6.10	− 31	− 3.9*	− 8.1, 0.4
Callao	4.25	5.93	40	0.4	− 7.1, 8.6
Ica	7.22	8.14	13	1.9	−4.4, 8.6
La Libertad	11.77	11.46	−3	− 3.0	−6.7, 0.7
Lambayeque	5.02	11.32	125	5.1	−5.1, 16.4
Lima	8.66	6.58	−24	−3.6*	−6.3, −0.8
Moquegua	16.74	10.87	−35	−8.5	−16.6, 0.3
Piura	10.26	5.97	−42	−4.3*	−7.7, −0.7
Tacna	9.45	16.20	71	3.5	−3.0, 10.5
Tumbes	4.86	2.26	−53	−8.1	− 18.7, 3.9
**Highlands region**					
Apurimac	5.61	12.84	129	6.9	−8.0, 24.3
Ayacucho	10.80	8.21	−24	−0.8	−9.4, 8.6
Cajamarca	8.81	9.62	9	−4.0	−11.9, 4.4
Cusco	10.63	11.94	12	1.3	−2.1, 4.9
Huancavelica	12.23	18.39	50	−0.5	−9.0, 8.8
Huanuco	25.70	22.67	−12	0.4	−3.3, 4.2
Junin	21.76	22.09	2	−0.3	−4.8, 4.3
Pasco	13.20	9.09	−31	−2.6	−13.9, 10.1
Puno	11.37	9.42	−17	2.3	−3.9, 8.9
**Rainforest region**					
Amazonas	30.04	23.53	−22	−2.4	−17.4, 15.3
Loreto	49.29	18.95	−62	−5.4	−13.1, 3.1
Madre de Dios	55.76	7.40	−87	−6.7	−19.6, 8.3
San Martin	19.35	17.46	−10	0.9	−3.2, 5.3
Ucayali	29.53	13.60	−54	−12.9*	−21.8, −3.2

* significantly different from 0 (*p* < 0.05)

APC, estimated annual percent change; CI: confidence interval

Table 2. Cervical cancer mortality rates (per 100,000 women-years), data availability, percent change, and estimated annual percent change and the corresponding 95% confidence intervals in Peruvian women between 2008 and 2017.

Table 3 shows cervical cancer mortality trends between 2008 and 2011 (Pre-Plan Esperanza) and 2012–2017 (Post-Plan Esperanza) in Peru and its regions. The mortality rates in Peru increased by 15% between 2008 and 2011 (except in the rainforest region) but declined considerably by 26% from 2012 to 2017. Similarly, the mortality rate in the coastal region increased by 28% between 2008 and 2011 but decreased by 30% between 2012 and 2017. In the highlands’ region, mortality increased by 13% between 2008 and 2011, but declined by 14% after 2012 and 2017, while the rainforest region showed a reduction of 17% between 2008 and 2011. Furthermore, 30% of a reduction between 2012 and 2017. None of these outcomes were statistically significant.

**Table 3:** Cervical cancer mortality trends (per 100,000 women-years), between 2008 and 2011 (Pre- Plan Esperanza) and 2012–2017 (Post- Plan Esperanza) in Peru and its regions

Geographical areas	Cervical cancer mortality rates (per 100,000 women-years)									
	Pre-Plan Esperanza					Post- Plan Esperanza				
	2008	2011	%change	APC	95%CI	2012	2017	%change	APC	95%CI
**Peru**	11.62	13.33	15	4.4	−1.1, 10.1	13.04	9.69	−26	−3.4	−9.1, 2.6
**Coast region**	8.61	11.01	28	8.0	−8.1, 27.1	10.63	7.40	−30	−4.5	−9.9, 1.3
**Highlands region**	13.58	15.31	13	4.4	−1.3, 10.5	15.99	13.69	− 14	−0.7	−8.0, 7.3
**Rainforest region**	34.16	28.33	−17	−6.9	−22.5, 11.8	25.83	17.98	−30	−4.7	−13.8, 5.5

Table 3. Cervical cancer mortality trends (per 100,000 women-years), between 2008 and 2011 (Pre- Plan Esperanza) and 2012–2017 (Post- Plan Esperanza) in Peru and its regions.

**Fig. 3:**
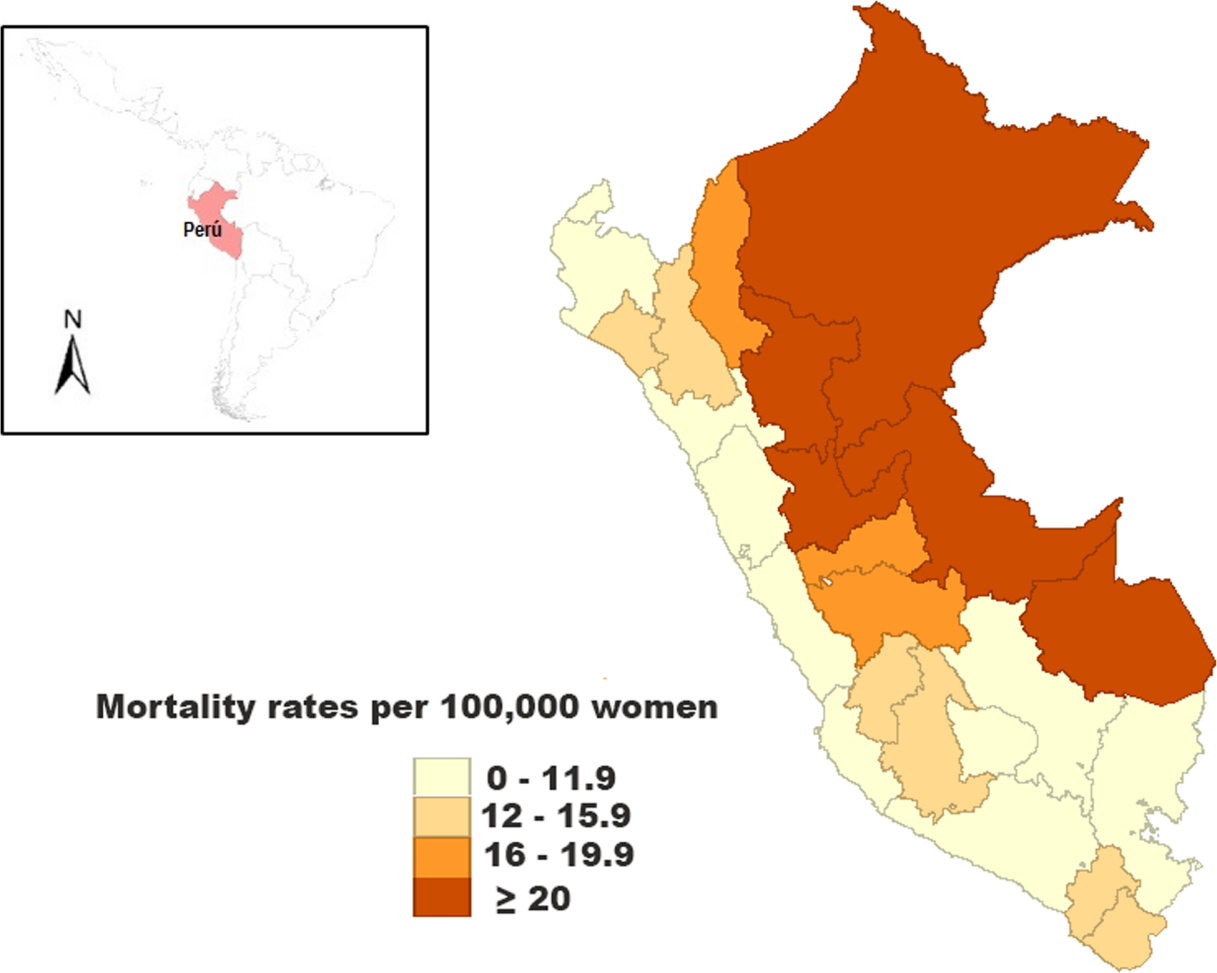
Spatial distribution of age-standardized mortality rates for cervical cancer in Peru from 2008 to 2017. Map created using GEODA version 1.14.0. Available at: https://geodacenter.github.io/index.html

**Fig. 4:**
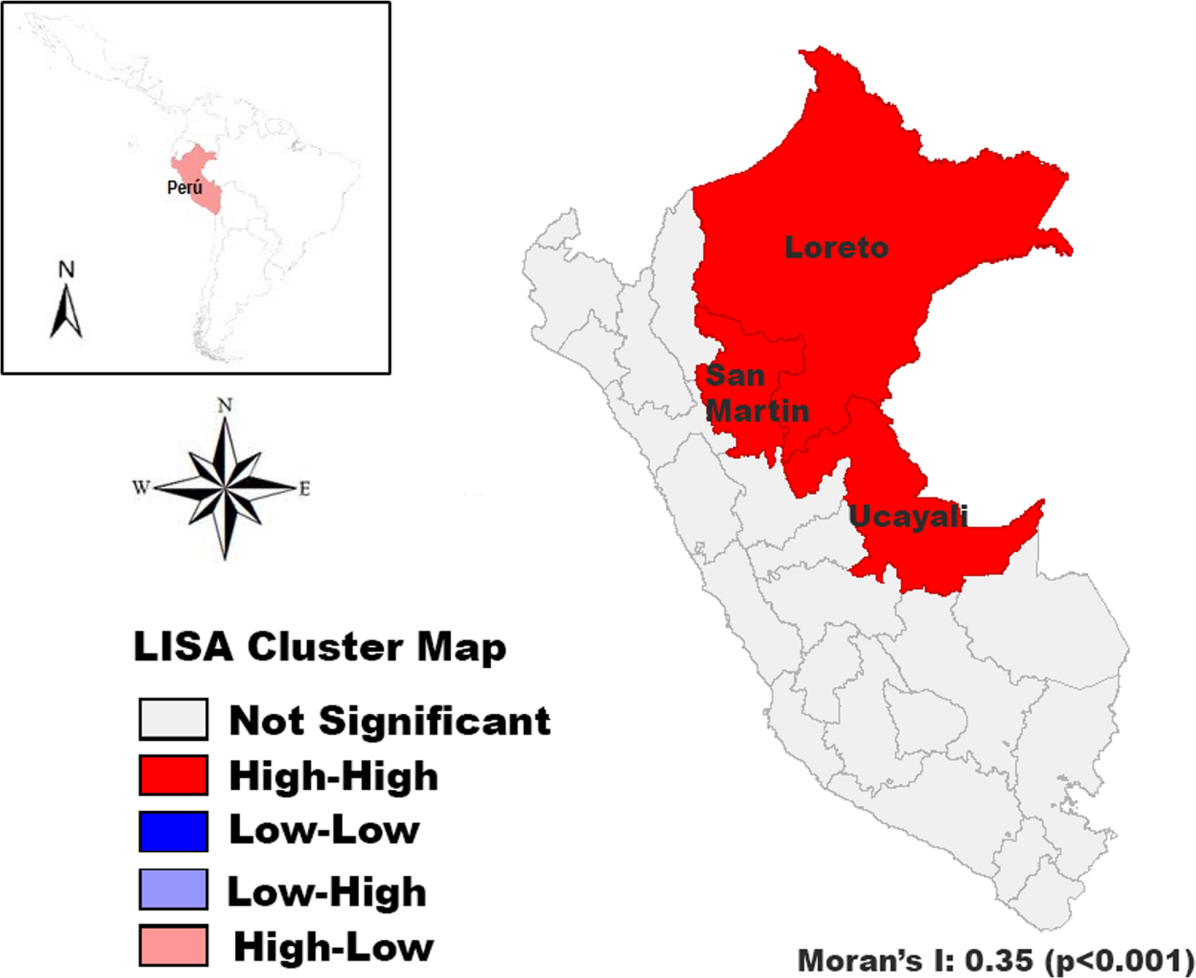
Spatial auto-correlation for cervical cancer mortality rates for the period 2008–2017. Map created using GEODA version 1.14.0. Available at: https://geodacenter.github.io/index.html

Figure 3 illustrates the spatial distribution for cervical cancer in Peruvian women between 2008 and 2017. The highest mortality rates were observed in the rainforest departments including Loreto, Ucayali, and Madre de Dios (ASMR ≥20 per 100,000 women-years), while the coastal departments had the lowest mortality rates. In reference to the spatial analysis and clustering, the mortality rates from 2008 to 2017 showed a positive spatial autocorrelation and significant clustering (Moran’s I: 0.35, *p* < 0.001). The departments with the highest cervical cancer mortality rates were in the neighboring North-East departments (Loreto, Ucayali, and San Martin) as shown in Fig. 4.

## Discussion

We found a decrease in cervical cancer mortality rates in Peru from 2008 to 2017. Despite these findings, Peru still has higher mortality rates compared to other Latin American countries such as Argentina (8.4), Brazil (7.3), Chile (6.0), Colombia (8.0), and Mexico (8.1) [26]. Between 2002 and 2018, cervical cancer mortality rates in Latin America decreased from 12.9 to 7.1 per 100,000 women [1, 27], with a further reduction in Brazil, Colombia, and Mexico. However, these mortality rates are higher than those of European countries [28–30], with rates of about 3 deaths per 100,000 women.

The downward mortality trends in cervical cancer could be rationalized by the continued efforts of the Peruvian government to reduce the burden of this disease. In 2007, “The concerted health plan” [31] was approved to reduce mortality by cervical cancer, promote prevention, screening, early diagnosis, and implement protocols to improve primary care delivery [31]. Later, in 2012, Plan Esperanza was implemented to expand health coverage and to provide comprehensive cancer health services to vulnerable populations, providing an early diagnosis of cancer (including cervical cancer) to reduce mortality rates [9]. In fact, Plan Esperanza has played an important role on decreasing the burden of mortality of cervical cancer in Peru. Nevertheless, the rainforest region has the highest cervical cancer mortality rates. There are several reasons are related to this outcome. The rainforest has a high density of indigenous women [32]. A study in Latin American indigenous women [33] showed a higher risk of death from cervical cancer, which could be explained by the lower detection rate of early-stage cervical cancer and barriers to access treatment [10, 33–35]. In addition, the socioeconomic disparities, lack of access to health care services, and low education in the rainforest region [36], have and continue to create a setting with a lack of knowledge, and poor education on cancer prevention, resulting in poor adherence to treatment and even rejection of screening [34, 35, 37]. These factors compromise the overall population health and prevent further reductions of mortality rates. As a result, there is a need to develop community programs for indigenous women and strengthen decentralization processes to obtain favorable mortality outcomes.

The coastal departments had the lowest mortality rates, and the overall region showed significant downward trends. The greater access to health care services and socioeconomic income of the population in this region further explains the findings of this study [3, 7]. Similarly, studies in Brazil and the United States reported a lower mortality rates in states with both higher human development indexes and economic incomes [38, 39]. Nevertheless, it is to be expected that rapid access to healthcare facilities and the availability of therapy would result in lower mortality trends in the coastal region.

Since the implementation of Plan Esperanza, the coverage for cervical cancer screening, including cytology (Papanicolaou smear or liquid-based cytology), cervical visual inspection with acetic acid, or cryotherapy, colposcopy, and molecular human papillomavirus tests has been made available in Peru [9]. Therefore, a higher rate of pre-neoplastical lesion detection in combination with access, prompted treatment in the coastal region, which will lead to a decrease in mortality rates.

However, this scenario is not feasible in the rainforest and highlands regions, due to the geographical barriers, are one of the principal limitations to accessing screening procedures and treatment. Despite the efforts to decentralize health care, this has not reached most of the population in the highlands. This explains the non-stable trend in these regions of our analysis, in contrast to the coastal and rainforest regions which showed a downward trend that has been more pronounced since the implementation of the Plan Esperanza (Table 3). Moreover, the remote rural areas of the coast departments generate high socioeconomic disparities and limitations in health care practice (e.g., supplies for Pap smears, poor standardization of procedures, inadequate training of health personnel, unavailability of appropriate therapies, high treatment costs, inefficient management of information systems, and delivery of results) [40]. Consequently, the preventive effect of prompt cancer detection and adequate treatment of patients in urban areas is hindered by poor health delivery in rural areas. These shortcomings affect preventive strategies and represent a limitation of decreasing mortality rates of cervical cancer in Peru.

## Strengths and limitations

This study is the first to report the regional mortality rates and trends by cervical cancer in the last decade in Peru. The results of this study provide a perspective of the current trends of cervical cancer mortality in the country and the impact of programs to reduce the burden of cervical cancer mortality such as the Plan Esperanza. The main limitation of this study is the underreporting of deaths, and the possibility that a proportion of cervical cancer deaths are registered as death by uterine cancer or of unspecified origin. In regions with poor health and communication systems, such as the department of Loreto, the rate of omission of death registration is as high as 78%. However, we corrected the underreporting and estimated reliable mortality rates of the female population across the country [16].

## Conclusion

In conclusion, although our results found decreasing in the mortality trends in the entire population, the overall rates by cervical cancer remain high, mainly in the rainforest region. Our results demonstrate the need for further development and the improvement of the current health care delivery systems for the most affected populations, and the provision of a wide range of health coverage to vulnerable women.

## Data Availability

The datasets generated and/or analyzed during the current study are available prior request in the following link: http://www.minsa.gob.pe/portada/transparencia/solicitud/

## Abbreviations

APC: Annual percent change
ASMR: Age-standardized mortality rates
